# New Scotch Lunacy Law

**Published:** 1857-10-01

**Authors:** 


### NEW SCOTCH LUNACY LAW.

THE following is a faithful abstract of the Act for the regulation of the care and treatment of Lunatics, and for the provision, maintenance, and regulation of Lunatic Asylums in Scotland :—

The preamble recites that it is expedient that the existing Acts (55 Geo. III. c. 69; 9 Geo. IV., c. 34; and 4 and 5 Vict., c. 60) should be repealed, and that more efficient provision should be made for the care and treatment of lunatics, and for the provision, maintenance, and regulation of lunatic asylums in Scotland.

SEC. 1 Repeals the existing Acts from and after the 1st January, 1858.

SEC. 2. All officers appointed under the existing Acts are to continue to act until reappointed or superseded, and all licences are to remain in force for the periods for which they were granted, or until revoked. All proceedings under the existing Acts are to be valid, excepting such as are expressly made void or affected by this Act; and all fees, charges, liabilities, and expenses are to be payable as heretofore.

SEC. 3 Interprets the meaning of the technical words and expressions used in the Act.

SEC. 4 Constitutes a "General Board of Commissioners in Lunacy for Scotland," to be appointed by the Crown, and to consist of one unpaid Commissioner, who shall be Chairman, and two paid Commissioners, at salaries not exceeding 1200*l.* each per annum, to be fixed by the Treasury. The Crown may appoint other unpaid Commissioners, not exceeding three in all, for any specified time, and fill up vacancies.

SEC. 5. The Board is to have an office at Edinburgh, and is to meet on the 1st of November next, and is to hold two General Meetings annually in March and November, three members to form a quorum. The Board may adjourn, and may hold special meetings. At all meetings the chairman is to have both an original and a casting vote.

SEC. 6. The Board may appoint two or more of their number as a committee, who may exercise all the powers of the Board, and are to report to the Board.

SEC. 7. Every Commissioner, before acting, is to take an oath of office and secrecy in the form prescribed by the Act.

SEC. 8. The Commissioners are not to derive any profit or emolument from their office, except as provided by the Act, nor are they to be personally responsible for anything done *bonâ fide* under the Act, and the paid Commissioners are to devote their whole time to the duties of the office.

SEC. 9. The Board, in addition to special powers, are to have the superintendence, management, direction, and regulation of all matters arising under the Act in relation to lunatics, and to public, private, and district asylums, and to every house in which a lunatic is kept under the order of the sheriff, and also the power of granting or refusing licences for private asylums, and of renewing, transferring, recalling, or suspending them; and are to make rules and regulations for all private and district asylums, and the officers and servants thereof, as well as their own officers and servants, and to enforce the same by forfeiture of licence and by recovery of penalties; but such rules are to be submitted to Parliament. And as regards existing *public* asylums (unless when otherwise specially provided by the Act), the Board are only to regulate their inspection and visitation, and to make rules in relation to the books and minutes, and the returns of the entries to be made to the Board.

SEC. 10. All *public* asylums endowed, founded, or established after the passing of this Act, and all additions to existing public asylums, are to be subject to the powers and provisions to which the Act subjects existing *public* asylums.

Sec. 11. The Board may investigate any case which they shall think it proper to inquire into, and, if it be necessary to obtain evidence, may, with the concurrence of the Lord Advocate or the Solicitor-General acting for him, summon any person to give evidence on oath, under a penalty not exceeding 30*l*. A form of summons is given in Schedule A.

Sec. 12 Provides for the payment of the reasonable expenses of witnesses.

Sec. 13 Provides for the appointment of a Secretary to the Board, at a salary not exceeding 500*l*. per annum, to be fixed by the Treasury, who shall give security: also for his removal, the appointment of a successor, the regulation of his duties by the Board, and security to be given by him.

Sec. 14. A return, to be prepared by the Secretary, is to be annually laid before Parliament, showing the number of orders for admission of lunatics into each asylum; the number of licences granted; the transfer of licences; the names of the superintendents; the number of patients, male and female, received into and discharged from each asylum, or removed or transferred from one asylum to another, classifying them as "cured," "relieved," and "unaffected by treatment," during the preceding year.

Sec. 15. The Secretary is to keep books and minutes of proceedings, accounts of moneys, and all charges and expenses under the Act; and an account is to be furnished annually to the Treasury, and audited, and the balance, if so directed, is to be paid into the Exchequer. An abstract of the accounts is to be laid before Parliament.

Sec. 16. The Board may appoint a clerk, at a salary not exceeding 150*l*. per annum, who shall give security, and perform such duties as the Board may require.

Sec. 17. The Board are to make general rules for the inspection and visitation of all asylums; and the two paid Commissioners are to visit, at least twice a year, every asylum and every house in which a lunatic is detained under order of a sheriff, and inquire into the condition of the lunatics—whether any coercion or restraint has been imposed—and are to record in the "Patients' Book" of the asylum, the state of health generally, mental and bodily, of the lunatics, what coercion or restraint has been imposed, and the cause thereof; and specially such particular cases as require remark; and also inquire into the management and condition of each asylum, as to its state of repair, heating, ventilation, cleanliness, supply of water, diet, and otherwise; and shall see that the patients do not exceed the proper number, and that the books and registers are properly kept. All persons connected with the management of the asylums, houses, and lunatics, are to disclose all particulars to the Commissioners, who are to record in a book all inspections, and communicate the same to the Board. The Commissioners may make any particular visitation or inquiry as they may think fit, by night or by day. A copy of all entries of the paid Commissioners, the sheriff, and justices of the peace, and of medical inspectors, in the Patients' Book, is to be transmitted to the Board by the superintendent of the asylum within eight days, under a penalty not exceeding 10*l*.

Sec. 18. The Commissioners are, once a year, or oftener, by day or night, to visit any prison in which there shall be, or be alleged or supposed to be, any lunatic, and make such inquiries as to the lunatics as they think proper or the Board may direct.

Sec. 19. The Commissioners are also, by day or night, to visit all poorhouses in which there may be any lunatic, and inquire whether the provisions of the law as to lunatics have been carried out in the parish, and also as to the dietary, accommodation, and treatment of the lunatics, and report in writing to the Board.

Sec. 20. The Board may, where necessary, take the assistance of such medical persons as may be required, and provide for the expense.

Sec. 21. If necessary, one of the Secretaries of State may appoint one or more medical persons (not exceeding two) to be Deputy Commissioners, at salaries not exceeding 500*l*. per annum each, who are to have the power of Commissioners; but no such appointment is to subsist after the expiration of five years from the passing of the Act.

Sec. 22. The Board is to exist five years only, from 1st January, 1858, after which time the two paid Commissioners are to become "Inspectors General in Lunacy for Scotland," subject to the orders of one of the Secretaries of State, and are to have the same power and duties as the Board—of visitation and inspection of all asylums, houses, prisons, poorhouses, and places in which any lunatic is kept—and are to perform such duties as the Secretary of State may prescribe. All notices required to be given to the Board are then to be given to the Inspectors General. Their salaries will then not exceed 1000*l*. per annum each.

Sec. 23. At the expiration of five years, the Secretary of State may empower the Inspectors General to exercise the powers of the Board in enforcing general regulations, providing accommodation for lunatics, and the citation and examination of witnesses, and generally any of the special powers of the Board; and from the same period, the power of granting licences is to be vested in the sheriff, but no licence is to be granted without a certificate from the Inspectors General, and no licence is to be continued if the Inspectors General report that it should be discontinued. The sheriff clerk is to receive and account for all fees and duties in respect of licences.

Sec. 24 Prescribes the oath of office and secrecy to be taken by every person appointed secretary, clerk, or medical or district inspector.

Sec. 25. The sheriff may at all times visit and inspect, either alone or with a medical person, every asylum or house within his jurisdiction in which a lunatic is kept under order of the sheriff, and inquire into its management, and the conduct of the officers and servants thereof, and is to insert in the Patients' Book any observations he may deem necessary.

Sec. 26. The justices of the peace of every county are to appoint, at quarter sessions, any three of their number to visit and inspect any asylum in such county, and insert in the Patients' Book their observations.

*Licences for Private Asylums—Orders—and Certificates.*

Sec. 27. All private asylums are to be licenced by the Board, and the licences are to be granted to the superintendent. All applications for licences, or for leave to transfer any licence, are to be made to the Board, accompanied by a statement of the name and qualification of the superintendent, and a plan of the house as the Board may direct; and the application must state the greatest number of lunatics of each sex proposed to be received, and any subsequent alteration in the house is to be shown on a plan when any application for the renewal of a licence is made.

Sec. 28. The licence to be granted by the Board must be according to the form in Schedule B, and bear a stamp of 10s., and may be for such period, not exceeding thirteen months, as the Board shall think fit. For every licence there is to be paid to the Secretary 10s. for every patient not being a pauper, and 2s. 6d. for every pauper patient; but no licence is to be delivered until 15*l*. shall be paid. If the licence be for less than thirteen months, the Board may reduce the payment proportionately.

Sec. 29. The Board, on refusing to grant renewal of licence, may, without further payment, continue the existing licence for a period not exceeding three months, during which time the asylum and the officers are to be subject to all the regulations as if the existing licence had been renewed.

SEC. 30. If a person having a licence shall become incapable of keeping, or be desirous to discontinue keeping the asylum, or shall die, the Board may, on application, transfer the licence; and in case of the death of any one or more of several persons to whom a licence has been granted, the licence remains in force to the survivors, or survivor.

SEC. 31. A payment of 5s. is to be made for every order granted by the sheriff for admission of a patient, not being a pauper, and for every pauper 2s. 6d. These sums are to be received by the sheriff clerk, and remitted by him to the Secretary, on the direction of the Board, under a penalty not exceeding 10*l*.

SEC. 32. The money received for licences and orders of admission, and for searches, are to be applied in payment of salaries and expenses of the officers, and any surplus is to be divided among the district boards, in the proportion of the sums raised by each district board. The money is to be lodged in a bank, and payments are to be made on the order of the Board, and accounts of expenditure are to be audited and passed.

SEC. 33. If the money received for licences, &c., be insufficient to pay salaries and expenses, the deficiency is to be paid by the Treasury.

SEC. 34. The sheriff is not to grant an order for the reception of a lunatic into an asylum or house unless upon a petition in the form of Schedule C, accompanied by certificates (dated within fourteen days of the date of the petition), in the form of Schedule D, of two medical persons, one of whom may be the medical superintendent or consulting physician of a public or district asylum; and no person is to be received or detained in an asylum as a lunatic without an order of the sheriff, dated within fourteen days prior thereto, or twenty-one days if granted by the Sheriff of Orkney or Shetland. But a person may be detained for not more than twenty-four hours, whose case is certified by one medical person to be a case of emergency.

SEC. 35. The medical person, in his certificate, must specify the facts upon which the opinion is formed, and distinguish facts observed by himself from facts communicated to him by others; and no person is to be received into any asylum or house under a certificate founded only upon facts communicated by others.

SEC. 36. An incorrect or defective order or medical certificate may be amended by the person signing the same, within fourteen days after the reception of the lunatic, but such amendment will have no force unless sanctioned by the Board.

SEC. 37. The superintendent of the asylum or house, after two clear days, and within fourteen clear days from the day on which the patient is received, is to transmit to the Board copies of the order, medical certificates, petition and statement on which such patient has been received, and a notice of such admission, and a report signed by the medical attendant of the asylum, or by the medical attendant of the lunatic in any house, in the form in Schedule F, under a penalty not exceeding 20*l*.; and the sheriff clerk is, within seven days after the granting of any order, to send to the Board a notice, stating the person making the application, the person to whom the order applies, the medical person granting the certificates, the sheriff granting the order, and the asylum to which it was addressed, under a penalty not exceeding 10*l*.

SEC. 38. No person is to grant a certificate, or statement, without having seen and carefully examined the patient, under a penalty not exceeding 50*l*.; and if any person shall wilfully and falsely grant a certificate to the effect of any person being a lunatic, he will be liable to a penalty not exceeding 300*l*., or to imprisonment for any period not exceeding twelve months.

SEC. 39. Any person convicted of receiving, concealing, detaining, or harbouring any lunatic in a house requiring a licence, but unlicensed, and any person convicted of so sending any lunatic; or any person convicted of receiving, concealing, detaining, or harbouring any lunatic in an asylum or house without an order, where such order is required, or notwithstanding an order for his liberation, or any person convicted of so sending any lunatic, shall be liable to a penalty not exceeding 100*l.*, or to be imprisoned for any space not exceeding twelve months.

SEC. 40. The Board may authorise search to be made as to whether any particular person has been confined as a lunatic within twelve months.

SEC. 41. A single patient cannot be received without the order of the sheriff and certificates, unless the house be the dwelling-place or private lodging of the lunatic. Any person receiving a single patient must, within seven days, transmit to the Board copies of the order, certificates, petition, and statement, and state the date of reception, situation of the house, and the name of the owner, occupier, and of the medical attendant, and send an annual medical certificate, and such patient is to be visited once a fortnight, or as the Board may direct, by a medical person, who shall enter in a book the date of each visit, and the condition of the lunatic's mental and bodily health; but this will not apply to a patient sent for a temporary residence not exceeding six months, under a medical certificate, in the form of Schedule G. Any one offending against these provisions will be liable to a penalty not exceeding 50*l.*, or imprisonment not exceeding three months.

SEC. 42. The Board may order the visitation and inspection of any house where a lunatic is detained by order of the sheriff, and in case of improper treatment may transfer the lunatic to another house or asylum, and the expense of maintenance will be chargeable on the lunatic's property, or on the party or parish legally bound for his support.

SEC. 43. Any person detaining a lunatic, although one of the family or a relative, in a private house, without an order by the sheriff, for more than a year, during any part of which coercion or restraint has been necessary, must intimate such detention to the Board, and transmit a medical certificate of the condition of the person so detained, and state the reasons rendering it desirable that such person should remain under private care. On suspicion of a person being so detained, the Board may apply to the sheriff, who may make inquiry; and if it shall appear that any person has been so detained, and it is expedient to remove the lunatic, the sheriff may issue a warrant for such removal. Any person infringing this provision will incur a penalty not exceeding 200*l.*, or imprisonment not exceeding three months.

SEC. 44. On the transfer of a licence from one house to another, the Board may authorize the transfer of the patients, without any fresh order from the sheriff or fresh medical certificates, provided due notice has been given to the persons on whose application the patients were confined. The superintendent is to give the Commissioner notice of such transfer of patients within eight days, under a penalty not exceeding 50*l.*

SEC. 45. Every asylum licensed for one hundred patients must have a resident medical attendant; and any asylum not having a resident medical attendant, if licensed for less than one hundred and more than fifty patients, must be visited daily by a medical attendant; and if licensed for fifty patients or less, must be visited at least twice a week by a medical attendant. The Board may decide that any asylum shall be visited at other times, but not oftener than once a day, and may require that any asylum licensed for more than fifty patients shall have a resident medical attendant.

SEC. 46. The Board may permit any asylum licensed for less than eleven patients to be visited at longer intervals, not exceeding once in every two weeks.

*Access of Friends and others to Lunatics.*

SEC. 47. The ministers of the parish in which the asylum is situated, or of the congregation to which the lunatic belongs, or any relative, or, in case of a pauper, any member of the parochial board liable to maintain the lunatic, may visit any such lunatic, subject to regulations to be approved by the Board. But the superintendent and medical attendant may, when it is expedient, refuse permission to visit, or accompany the permission with conditions. Any complaint must be made to the Board, whose decision must be final. Every refusal is to be entered in the register of the asylum, and a copy sent to the Board within two days.

Sec. 48. The Board may give written orders of admission to visit patients, either with or without any restrictions, and any superintendent or keeper refusing admission will be liable to a penalty not exceeding 20*l.*

*District Asylums.*

Sec. 49. With a view to the erection of asylums for pauper lunatics, Scotland is to be divided into the districts described in Schedule H; but the districts may be varied by the Board, on the application of any prison board of the county interested.

Sec. 50. "District boards" are to be elected annually by the prison boards, the number of members and the times and places of meeting to be fixed by the general board. Vacancies are to be filled up at the next meeting of the prison board. The district boards may adjourn and may appoint committees of their number, to whom they may delegate all or any of their powers. Their meetings are to be conducted as meetings of a prison board.

Sec. 51. The Board is to ascertain the existing accommodation for pauper lunatics in each district, and, where it is insufficient, to require the district board to procure plans and estimates for providing the requisite accommodation, and to report to the Board.

Sec. 52. The Board, on approving the plan, may require the district board, within two years, to provide a suitable district asylum for pauper lunatics, for which purpose they may purchase the necessary land.

Sec. 53. District asylums, unless by their constitution otherwise vested, are to be vested in the district boards; and if they become insufficient, the Board may require the district board to increase the accommodation.

*Assessments.*

Sec. 54. The expense of providing district asylums, and the first year's expense of maintaining the establishment, and the expense of keeping them in repair, is to be ascertained, and raised by an assessment on the rental of the property in the district.

Sec. 55. The amount assessed for each district is to be remitted within eight months to the district board, who are to apply the same in defraying the expenses of providing the asylum, and also the first year's expenses of the officers, so long as the funds of the asylum are inadequate.

*Special Arrangements.*

Sec. 56. Any property or money held in trust for the establishment of an asylum for a county or parish may be contributed towards establishing a district asylum, and the assessment upon such county or parish will be relieved to the extent of the contribution

Sec 57. Any county or parish having an asylum available for paupers, and transferring the same to the district board, will be relieved to the extent of its value from assessment.

Sec. 58. If in any district there be an asylum wherein another district, or a parish in another district, has a right to accommodation, the Board of the district in which the asylum is situated may purchase such right to accommodation, and the purchase-money is to go in reduction of the assessment on the district or parish to which such right belonged.

Sec. 59. Where there is an existing asylum in any district, having sufficient accommodation for the pauper lunatics of the district, the district board, instead of erecting an asylum, may agree with the proprietor for the reception and maintenance of the pauper lunatics, and the portion of the asylum set apart for the purpose is to remain under the management of the proprietor, subject to the inspection and regulations of the Board.

Sec. 60. The trustees of the Crichton Royal Institution for Lunatics, at Dumfries, are to receive in that asylum, or in the Southern Counties Asylum, the pauper lunatics of the counties Dumfries and Wigtown, and the stewartry of Kirkcudbright, upon the terms prescribed by the Act for pauper lunatics.

*Borrowing Money.*

Sec. 61. The district board may borrow money for the purposes of the Act at five per cent., on the security of the assessments, in the form in Schedule K, No. 1, a copy of which is to be transmitted to the Secretary, and registered. The security may be transferred by endorsement in the form in Schedule K, No. 2.

Sec. 62. The district board may borrow money from the Public Works Loan Commissioners.

Sec. 63. The district board is annually to pay all interest due on money borrowed, and to set apart not less than one-thirtieth part of the total sum borrowed until the whole be paid. The order in which the sums are to be paid off is to be determined when the same are advanced. Accounts are to be kept.

Sec. 64. Every district board borrowing money is to provide that the whole, principal and interest, shall be discharged within thirty years.

Sec. 65. Persons lending money on the security of the assessments are not bound to require proof that the provisions of the Act have been complied with, and the validity of the security is not to be questioned.

Sec. 66. The district board may pay off money already borrowed, with consent of the lender, and for that purpose borrow other money, but all principal and interest must be paid off within thirty years from the time of first borrowing the same.

Sec. 67. The district boards are annually, and whenever required by the Board, to transmit to them a detailed report and statement of all principal and interest falling due, for which the Board is to provide, in ascertaining the sum to be raised in each district.

Sec. 68. District asylums (except asylums mentioned in section 59) are, when completed, to be under the charge of the district boards, who are to appoint the necessary officers and servants, and fix their salaries, and see to the management and discipline of the asylum.

Sec. 69. On completion of the district asylum, the district board is to give notice in the *Edinburgh Gazette* on what day the asylum will be open for the reception of lunatics, which must be not less than a week subsequent to the notice.

Sec. 70. The district board is to appoint one or more medical inspectors, and fix the fees to be paid to them, with the approbation of the Board. The inspectors are to visit all asylums at the times required by the district board, Board, or sheriff, and to enter in the Patients' Book the condition of the asylum and of the lunatics, and the particulars of any case requiring remark. Where more than one inspector is appointed, it is not necessary that more than one should be a medical person.

Sec. 71. No unqualified medical person is to practise, or be employed, or to grant a certificate under this Act; and no medical person having any pecuniary interest in any asylum, or whose father, brother, or son shall be superintendent of an asylum, is to practise, or be employed, or to grant a certificate under this Act, with reference to such asylum, under a penalty of 50*l*., or imprisonment not exceeding three months. But the medical officer of a district asylum may grant certificates with reference to any lunatics of the district to which the asylum belongs.

SEC. 72. In case of refusal or neglect on the part of commissioners of supply, magistrates or others to carry the Act into execution, so far as it applies to them; or in case of any obstruction, the Board may apply by petition to the Court of Session.

SEC. 73. The payment to the district board for each pauper lunatic is to be such sum per week, by such periodical payments as the district board shall fix with the approbation of the Board, which is to be applied in payment of all expenses and salaries. If the payment be insufficient, the necessary additional charge is to be made for each pauper lunatic; or the Board may, where the burden on the parishes would be unreasonable, fix a maximum rate to be charged for each pauper lunatic, and the balance will be raised by assessment on the district.

SEC. 74. The district boards are to keep regular books, and accounts of receipts and expenditure, and minutes of proceedings. Three members are to form a quorum. An account of all assessments and other moneys levied, and of the application thereof, is to be transmitted to the Board half-yearly.

SEC. 75. Every pauper lunatic in a district asylum is to be chargeable to the parish of his legal settlement at the time the order for his reception was granted, and that parish will be deemed his residence.

SEC. 76. The parish of the settlement of a pauper lunatic is to pay all expenses of placing him in a district asylum and his maintenance there; and the sheriff of the county from which he was removed may fix the amount of the expenses, which will be recoverable by summary process.

SEC. 77. The expenses of removal and maintenance of any lunatic are to be defrayed out of his estate: or if he has no adequate estate, and such expenses be not borne by the lunatic's relatives, he is to be treated as a pauper lunatic, and his expenses are to be paid by the parish of his settlement.

SEC. 78. If the parish of the settlement of a pauper lunatic cannot be ascertained, and if the lunatic has no means of payment, nor any relations liable, the expenses are to be paid by the parish from which he was taken; but they will be recoverable at any time when it shall appear that they are legally chargeable to any other party or parish. The sheriff is to certify the amount, which will be recoverable by summary process. But the parish of settlement will only be liable, on receiving written notice, for the expenses incurred subsequent to the notice and for the year preceding.

SEC. 79. Parties interested in the expense of maintaining a pauper lunatic, and his relatives, on warrant of the sheriff, are to have free access to see him.

SEC. 80. Where a district asylum has more than sufficient accommodation for the pauper lunatics of the district, the district board may, with the sanction of the Board, receive other lunatics, whether paupers or not, and take an undertaking for the payment of all expenses of maintenance, and of his burial in case of his death, and for his removal on notice. Lunatics not being paupers are to have the same accommodation as pauper lunatics.

### *Property of Lunatics.*

SEC. 81. Where the property of a lunatic is not under the management of a judicial factor, and it is supposed that such property or the income is not applied for his maintenance, the Board, or the Accountant of the Court of Session, is to report to the Lord Advocate, who may apply to the Court of Session for an investigation and the appointment of a judicial factor, and the application of the property, or the income thereof, for the lunatic's maintenance; and after investigation the Court may appoint a judicial factor, and take any other measures for the benefit of the lunatic.

SEC. 82. Where the property of a lunatic, though under the management of a judicial factor, does not appear to be properly applied for his maintenance, the Board, or the Accountant of the Court of Session, is to report to the Lord Advocate, who may apply to the Court of Session for an investigation, and the Court may make such orders and take such proceedings as may be expedient.

SEC. 83. The expenses of any inquiries relating to a lunatic's property is to be chargeable against such property.

SEC. 84. The caution or security to be taken for any judicial factor of a lunatic is to require the approval of the Accountant of the Court of Session; and where any caution received before the passing of this Act is considered insufficient, the accountant is to inquire into it, and where necessary require additional caution, and if the same be not provided he is to bring the matter under the notice of the Court of Session.

*Dangerous and Criminal Lunatics.*

SEC. 85. In case of a lunatic apprehended and charged with assault, or other offence inferring danger, or a dangerous lunatic at large, the sheriff may, on an application accompanied by a medical certificate, commit the lunatic to safe custody, when notice is to be given by advertisement of an inquiry into his condition, and on being satisfied that he is a dangerous lunatic the sheriff is to commit him to an asylum, at the charge of the person or parish liable for his maintenance, where he will be detained until cured, or until caution or security shall be given for his safe custody.

SEC. 86. If application be made to the sheriff respecting a pauper lunatic having a settlement in another county, the sheriff may proceed, or at once send the application and the lunatic in safe custody, to the sheriff of such other county, who may proceed upon the application.

SEC. 87. Where a person indicted for a crime is found insane, the Court is to direct a finding to that effect to be recorded, and is to order the lunatic to be kept in strict custody until her Majesty's pleasure shall be known, and her Majesty may make an order for his safe custody.

SEC. 88. Where evidence is given that a person indicted for a crime or offence was insane at the time of committing such crime or offence, and he is acquitted, the jury will be required to find specially whether such person was insane at the time of committing such crime or offence, and whether such person was acquitted on account of insanity; and if so, the Court is to order such person to be kept in strict custody until her Majesty's pleasure be known, and her Majesty may give an order for his safe custody.

SEC. 89. If any person, while imprisoned under sentence of death or other sentence, or under any criminal charge or civil process, shall appear insane, the sheriff is to inquire, with the aid of two medical persons, into his insanity; and if they certify that he is insane, the Secretary of State may by warrant direct his removal to an asylum; and all persons removed from prison to an asylum by reason of insanity, are to remain in confinement in such asylum until two medical men certify to the Secretary of State that such person has become of sound mind; whereupon the Secretary of State may by warrant direct his removal to prison, or his discharge, if his period of imprisonment shall have expired.

SEC. 90. In remote parts of the country, where necessary dangerous or pauper lunatics may, under warrant of a justice of the peace of the county, granted on sworn information, be sent for safe custody to the nearest town in which a sheriff or sheriff substitute shall reside, and the person having the custody of the lunatic must forthwith obtain the requisite medical certificates and order of the sheriff, when the case will be dealt with in the ordinary way.

SEC. 91. On the application of the Procurator Fiscal, or any of the Commissioners, accompanied by two medical certificates that the asylum is unsuitable for the confinement of the lunatic, the sheriff may order his removal to some other asylum or house; but notice of such application must be given to the person at whose instance the lunatic was confined, or if he be dead, or cannot be found, to his nearest known relative, and the expenses are to be defrayed by the party or parish liable for his maintenance.

SEC. 92. On production of the certificate of two medical persons, approved by the sheriff, of the recovery of the lunatic, or that he may be set at large, and the sheriff's order for his liberation, the superintendent of the asylum is to liberate him. The Board may also, on being satisfied by two medical certificates, order the liberation of such person. But previous to the liberation of any person, eight days' notice in writing must be given to the person at whose instance the lunatic was detained, or, in his absence, to the lunatic's nearest known relative, and in case of a pauper, to the party or parish maintaining him. The particulars and date of all cases of removal or liberation, and the authority for the same, must be entered in the register; and when a lunatic is discharged as incurable, such discharge must be entered, with the place to which, and the person under whose care he has been sent. Copies of all entries must be sent to the Board within two days.

SEC. 93. No such removal or liberation of a lunatic detained under the sentence of a court, shall take place without the authority of the court, or a warrant from the Secretary of State; and if by the expiration of the period of confinement awarded by the sentence, the lunatic would be entitled to discharge, but he is then uncured, he may be detained or removed to another asylum, upon two medical certificates and the order of the sheriff.

SEC. 94. On the release from an asylum of any person considering himself to have been unjustly confined, a copy of the order, petition, statement, and certificates upon which he was confined is at his request to be furnished to him by the clerk to the Board without fee.

SEC. 95. Every pauper lunatic is to be sent to the asylum for the district in which the parish of his settlement is situated, unless, under special circumstances, the parochial board, with consent of the Board, dispense with his removal, and provide for him in such other manner as the Board may sanction.

SEC. 96. A "register of lunatics" must be kept in every asylum, setting forth all particulars relating to every lunatic, in the form of Schedule I., and a copy transmitted to the Board, under a penalty not exceeding 20*l.*

SEC. 97. A register of deaths is to be kept in every asylum, setting forth the time and cause of death, and the duration of the disease, prepared and signed by the medical attendant in the form in Schedule J; and a certified copy is to be sent to the Board within three days, and also to the party or parish maintaining the lunatic, and to the person on whose application he was confined, under a penalty not exceeding 50*l.*

SEC. 98. A general register of all lunatics taken care of under this Act is to be prepared under the direction of the Board, who may at their discretion give or refuse information; and no inspection of the register is to take place without their written authority, under a penalty not exceeding 50*l.*

SEC. 99. Any officer of an asylum, or other person having charge of a lunatic, guilty of maltreating or neglecting such lunatic, will be liable to a penalty not exceeding 100*l.*, or imprisonment not exceeding six months, without prejudice to any action for damages. Where the maltreatment amounts to an assault, the public prosecutor may at his discretion prosecute for the assault or for the offence under this Act.

SEC. 100. The Lord Advocate may at all times examine and inspect all books, registers, minutes, proceedings, reports, returns, accounts, and documents in the possession of the Board, who are to afford him all information he may require.

SEC. 101. Any person wilfully making false statements, or refusing to give information required of him by this Act, will be liable to a penalty not exceeding 100*l*., or imprisonment not exceeding twelve months.

SEC. 102. The Board is to report annually to the Secretary of State as to the condition and management of all asylums.

SEC. 103. Orkney and Shetland, with their respective dependencies, are to be taken as separate counties for the purposes of the Act.

SEC. 104. The Home Secretary may direct special visitation and examination of lunatics, and a report thereon; and persons having the care of such lunatics are to facilitate the execution of the order.

SEC. 105. The Home Secretary may employ the Board, or any person, to make special inquiry into the state of any asylum, and to report to him; and may authorize remuneration and payment of travelling and other expenses.

SEC. 106 Points out the mode in which penalties and forfeitures are to be recovered.

SEC. 107. Penalties recovered in respect of any public or private asylum are to be applied towards the general expenses of the Board; and penalties recovered in respect of any district asylum are to be applied in payment of the expenses of the district asylum. No proceedings for recovery of a penalty or forfeiture can be commenced more than six months after the commission or discovery of the offence.

SEC. 108. Proceedings for recovery of penalties or forfeitures are not to be set aside for want of form, nor to be subject to appeal.

SEC. 109. Powers granted to sheriffs by this Act are to be in addition to, and without prejudice to their powers by law.

SEC. 110. The prison board of any county may, within six months, by resolution duly communicated to the Board and advertised, constitute such county into a separate district.

SEC. 111. The Board, for five years from 1st January, 1858, and the Inspectors General afterwards, may enforce the provisions of this Act by summary application to the Court of Session or to the sheriff.

SEC. 112. Inspectors of the poor, within seven days after discovery of a pauper lunatic, are to notify to the chairman of the parochial board and to the Commissioners in Lunacy the name, residence, state and condition of such lunatic, and the steps he has taken for his care and custody, under a penalty of 10*l*.

SEC. 113 Repeals so much of the Poor-law Act (8 and 9 Vict. c. 83) as empowers the Board of Supervision in certain cases to provide for the removal of insane and fatuous poor persons to lunatic asylums.

SEC. 114 Enacts that the assessing clauses of the Act shall not extend to the county of Shetland.

